# Tumor-infiltrating lymphocyte therapy in triple-negative breast cancer: from mechanistic exploration to clinical translation

**DOI:** 10.3389/fimmu.2026.1741333

**Published:** 2026-02-27

**Authors:** Yongxi Wang, Zhe Zhang, Zhuo Chen, Xiaoyun Xie, Fang Wang

**Affiliations:** Department of Breast Surgery, First Affiliated Hospital of Zhengzhou University, Zhengzhou, China

**Keywords:** immunotherapy, predictive biomarkers, TIL therapy, TILs, TNBC

## Abstract

Breast cancer is a common malignancy among women, with triple-negative breast cancer (TNBC) representing a subtype with poor prognosis. Due to the lack of expression of targetable receptors, traditional hormone therapy and HER2-targeted therapy are ineffective against TNBC. Moreover, TNBC typically exhibits more aggressive biological behavior, with a high propensity for recurrence and metastasis, further exacerbating its poor prognosis. While chemotherapy remains the primary treatment modality, its efficacy is limited, and patients readily develop resistance. Consequently, exploring novel therapeutic strategies and targets is crucial for improving the prognosis of patients with TNBC. Tumor-infiltrating lymphocytes (TILs) are promising prognostic and predictive biomarkers of TNBC. Multiple studies have demonstrated that a higher number of TILs in early-stage TNBC is correlated with favorable outcomes. Furthermore, clinical trials have demonstrated that TIL therapy is effective in solid tumors. This review outlines the current understanding of the TIL role in TNBC, elucidates the mechanisms and clinical efficacy of TIL therapy, and discusses future research directions and challenges for TILs.

## Introduction

### Clinical challenges of triple-negative breast cancer

TNBC is characterized by the absence of estrogen and progesterone receptors, as well as being HER2-negative (human epidermal growth factor receptor 2). Compared to hormone receptor-positive and HER2-amplified tumors, TNBC exhibits greater heterogeneity, accounting for 15%–20% of all breast cancer cases (1). TNBC has a higher recurrence rate and is associated with poorer progression-free survival (PFS) and overall survival (OS) (2, 3). The five-year OS for early-stage TNBC is only 77%, compared to 91% for all breast cancer (American Cancer Society 2022). Due to the lack of therapeutic targets, surgical intervention and chemotherapy remain the primary treatment for TNBC (4). Recently, immunotherapy has gradually gained prominence, with immunotherapy and antibody-drug conjugates demonstrating significant efficacy in this subtype. However, survival following metastatic relapse remains shorter compared to other breast cancer subtypes, and the treatment options are more limited.

### Basic concepts of TME and TILs

Tumors can recruit numerous other cells into their surrounding environment, consequently forming a tumor microenvironment (TME). The TME encompasses all the surrounding elements of a tumor, including blood vessels, fibroblasts, immune cells, signaling molecules, exosomes, and the extracellular matrix. As demonstrated in other cancers, TME plays a central role in tumor progression, immune evasion, and resistance to conventional anti-cancer therapies, consequently influencing disease progression (5, 6). TILs refer to a heterogeneous population of lymphocytes residing within tumor parenchyma and tumor stroma (7) and play a crucial role in the immune response by regulating cancer cell growth and proliferation. TILs constitute an “immune task force” that infiltrates tumor tissue. Comprising diverse lymphocyte types that collaborate and constrain one another, they collectively influence tumor progression. TILs primarily comprise T lymphocytes, especially CD8 cytotoxic T lymphocytes (CTLs) and CD4 helper T cells, although other immune subsets, including B cells and natural killer (NK) cells, may also be present (8, 9).

## TILs in TNBC

### Immunological characteristics of TNBC

TNBC is considered the most immunogenic breast cancer subtype because of its high tumor mutation burden, relatively abundant TILs, and elevated PD-L1 expression rates (10, 11). This primarily stems from the genomic instability characteristic of this tumor type, which generates a greater number of neoantigens (12). Despite the high immunogenic potential of TNBC, numerous immunosuppressive factors coexist. These include the infiltration of immunosuppressive cells, alongside the expression of immunosuppressive cytokines (TGF-β and IL-10), and the expression of other immune checkpoint molecules (LAG-3 and TIM-3) (13). These factors collectively create an immunosuppressive environment that limits the ability of the immune system to mount an effective response. This characteristic renders immune checkpoint inhibitors (ICIs) combined with chemotherapy a significant therapeutic option for advanced and early-stage high-risk TNBC, driving exploration of multiple combination treatment strategies.

Nevertheless, the heterogeneity of TNBC remains a formidable challenge. In recent years, increasing research has demonstrated that TILs are associated with TNBC progression. A review indicates that within TNBC TME, the enrichment of certain TIL subtypes is correlated positively with a favorable prognosis (14).

### Biological characteristics of TILs in TNBC

T-cell infiltration is more pronounced in TNBC than in other breast cancer subtypes (15). Based on CD expression on the cell surface, T cells are categorized into two primary groups: CD4^+^ T cells and CD8^+^ T cells (16). CD8^+^ T cells constitute the predominant lymphocyte population in infiltrating breast cancer, forming a critical line of defense against antitumor immunity. These cells recognize specific antigen peptides on the surface of tumor cells and release substantial quantities of IFN-γ, granzyme B, and perforin to destroy tumor cells. Previous studies have demonstrated that high CD8^+^ T cell expression is significantly associated with improved survival in patients with TNBC (17). CD8^+^ TILs in breast tumors are predominantly effector memory cells, express checkpoint molecules PD-1 and TIGIT. Despite PD-1 expression, CD8^+^ TILs maintain cytokine production capacity and retain the ability to degranulate and kill target cells. Thus, PD-1 CD8 TILs from breast cancer retain polyfunctionality (18). Tissue-resident memory T (T_RM_) cells constitute a non-recirculating CD8 T cell population that is permanently resident within peripheral tissues and conveniently mediates regional tumor surveillance. CD8^+^ TILs with a T_RM_ cell phenotype are associated with a favorable prognosis in patients with TNBC. T_RM_ could also provide local immune protection against tumor rechallenge and a T^+RM^ gene signature extracted from tumor-free tissue was significantly associated with improved clinical outcomes in TNBC patients treated with immune checkpoint inhibitors (19). CD4^+^ T cells, central to immune function, release anti-tumor cytokines and stimulate more immune effectors (20). Naive CD4^+^ T helper (Th) cells commonly differentiate into several effector cells, including Th1, Th2, Th17, and regulatory T cells (Tregs) (21). Th1-secreted interferon gamma (IFN-γ) not only induces the activation of signal transducer and activator to promote the differentiation of more Th1 cells but also recruits CD8^+^ T cells, NK cells, and other immune cells to enhance anti-tumor immunity, while Th2 cells with tumor-promoting effects mainly depend on the induction of interleukin (IL)-4 for differentiation. Importantly, IFN-γ inhibits the differentiation of Th2 cells, and IL-4 inhibits the differentiation of Th1 cells, thereby mutually restricting each other to maintain balance in the tumor. IFN-γ and IL-4 inhibit the development of Th17 cells (22, 23). Tregs promote tumor growth and metastatic seeding in patients with breast cancer. TNBC exhibits higher Treg cell infiltration, and deregulation of plasticity between Treg and Th17 cells creates an immune regulatory framework that enables tumor progression (24, 25).

**Figure 1:**
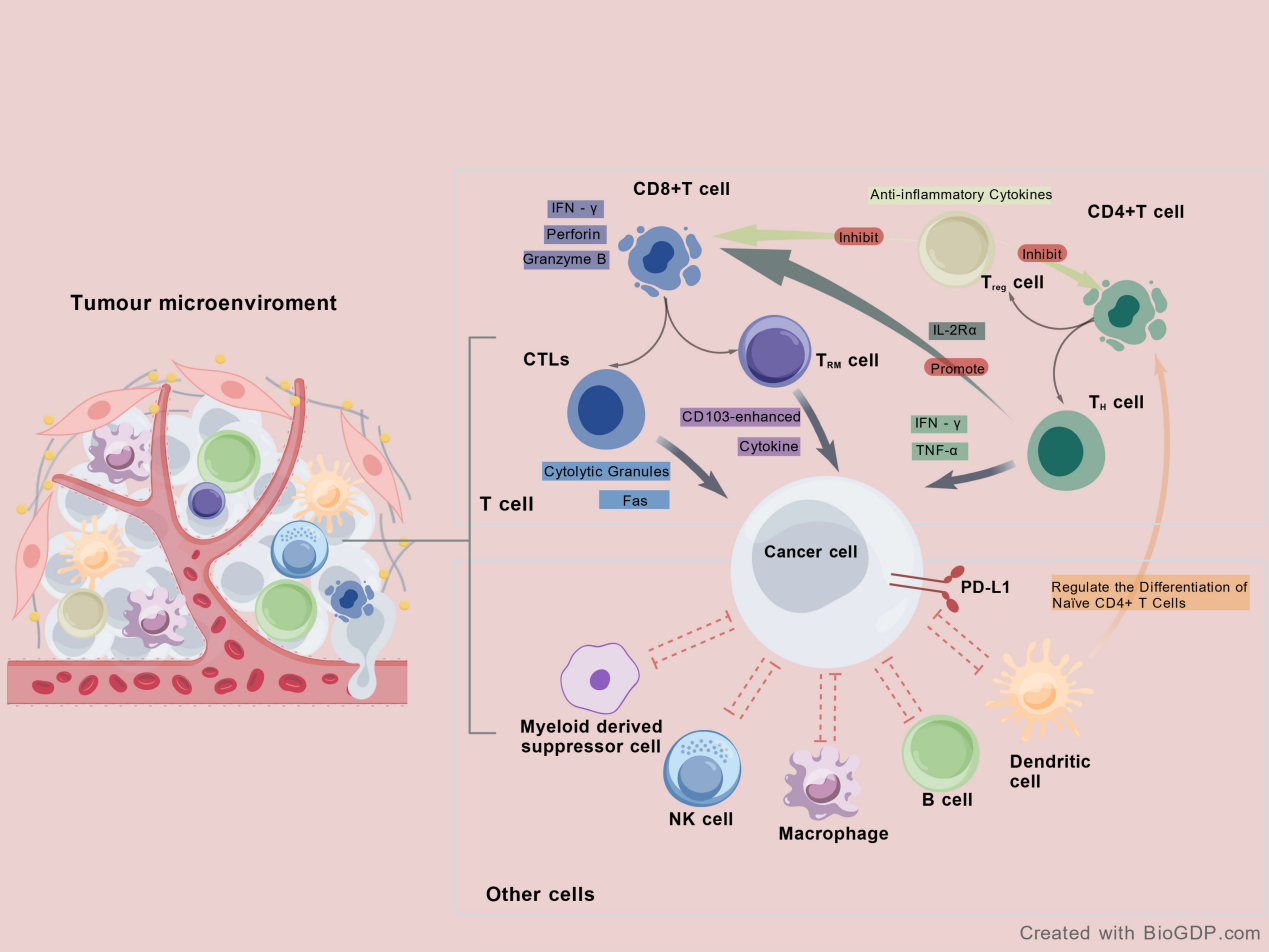
The primary immune cell types identified within the TME include macrophages, polymorphonuclear cells, mast cells, natural killer cells (NK), dendritic cells, and T and B lymphocytes. CD8^+^ T cells recognize specific antigen peptides on tumor cell surfaces and release substantial quantities of IFN-γ, granzyme B, and perforin to destroy tumor cells. CTLs constitute a class of T lymphocytes possessing cytotoxic functions against tumor cells. These effector T cells develop from activated naïve CD8^+^ T cells and exert tumor-killing capabilities. The polarized structure between CTLs and their target cells underpins the cytotoxic function of CTLs, ultimately leading to target cell death. CTLs primarily exert their tumor-specific killing activity through cytotoxic granules (CG) release. Additionally, secreting cytokines, CTLs exert cytotoxic activity by activating the Fas apoptosis pathway in target cells. Tissue-resident memory T (TRM) cells may mediate tumor protection by promoting tumor-immune equilibrium through cytokine secretion and/or through CD103-enhanced tumor cell killing. Within the tumor immune microenvironment, CD4^+^ T cells serve as helper T cells. These can be subdivided into distinct subsets that participate in immune regulation. DCs regulate the differentiation and polarization of naïve CD4+ T cells into various helper T cell subsets, such as Th1, Th2, Th9, Th17, Tfh, and Treg cells. Activated Th1 cells secrete two crucial Th1 cytokines: IFN-γ and TNF-α, mediating direct tumor growth inhibition by inducing cell death, senescence, cell cycle arrest, and proliferation arrest. Th1 cells are known to secrete IL-2, a cytokine that activates IL-2Rα expression and promotes proliferation in CD8+ T cells, thereby enhancing antitumor responses. Furthermore, Th1 cells can regulate B-cell-mediated antibody production and NK cell-dependent antibody-dependent cellular cytotoxicity (ADCC) in cancer. CD4+ regulatory T cells (Tregs) play a role in cancer progression by suppressing CD4+ and CD8+ effector responses through the anti-inflammatory cytokines secretion such as IL-4, IL-10, and TGF-β, and by exerting various other immunosuppressive effects on TME.

The dynamic interplay between pro-tumor and anti-tumor immune responses, generated by various immune cells within the TME, dictates the growth potential of the tumor and shapes cancer progression and metastasis **Figure 1**.

### Standardized assessment of TILs in TNBC

TILs have been described as biomarkers of the host immune response to tumors, exhibiting prognostic properties (26). The International Immunotherapy Biomarker Working Group has proposed a standardized method for quantifying TILs in solid tumors to enhance consistency and reproducibility in scoring (27, 28). Visual assessment is highly subjective and poorly reproducible, especially in complex scenarios such as heterogeneous distribution, residual lesions post-treatment, and stromal region delineation, where pathologists of varying experience may yield inconsistent judgments. The International Tumor Immunology Working Group (ITWG) has conducted a comprehensive analysis of the benefits of computational TIL assessment, while emphasizes critical barriers to clinical translation. To overcome these challenges, assessing TILs is rapidly advancing towards standardization, automation, and digitalization. Denkert et al. provided compelling evidence that software-guided image evaluation can enhance inter-observer consistency (29, 30). A study demonstrated that digital image analysis (DIA) can reliably assess TIL density in hematoxylin and eosin (HE) sections of core needle biopsy (CNB) specimens from breast cancer. Training and fine-tuning DIA software to recognize stromal elements and mononuclear cells yielded data on TIL density that exhibited high concordance with visual assessments by human observers (31). Furthermore, recent research has developed artificial intelligence-based reference cards (RCs) and introduced a field-of-view-based RC-assisted interpretation method, facilitating large-scale clinical implementation and validation. Studies indicate that the intraclass correlation coefficient (ICC) for AI-assisted assessment (AI-AS) reaches 0.966 (excellent), significantly surpassing the 0.730 (moderate) for traditional visual assessment (VA) and the 0.834 (good) for reference card-assisted assessment (RC-AS). The AI model also demonstrates outstanding efficacy in predicting pathological complete response (pCR), achieving an area under the curve (AUC) of up to 0.937 (32). Traditional visual assessment is being revolutionized and augmented by digital, automated AI approaches, promising to significantly enhance the accuracy, reproducibility, and efficiency of evaluations. Concurrently, multi-immunofluorescence technology offers a powerful tool for exploring the immune microenvironment through its multi-target, single-cell resolution, and spatial information analysis capabilities (33).

These technological advances collectively propel the widespread application of TILs as reliable biomarkers for prognostic stratification, treatment decision-making, and efficacy prediction in TNBC, ultimately facilitating more precise personalized therapies. In short, standardized TIL assessment optimizes individualized prognosis prediction and provides a basis for formulating immunotherapy strategies. Future studies should explore the potential of TIL-guided treatment de-escalation or immunotherapy combination regimens in high-risk patients.

## Prognostic and predictive value of TILs in TNBC

### In early-stage TNBC (received neoadjuvant chemotherapy)

Several previous pooled analyses have examined the prognostic role of TILs in patients with early-stage TNBC. One of these pooled analyses indicated that the prognostic effect exhibits a linear relationship with the logarithm of breast cancer event risk (34) for every 10% increase in TILs, invasive recurrence or death risk, distant recurrence or death, and death alone were significantly reduced by 13% (95% CI 9% to 17%), 17% (95% CI 12% to 22%), and 16% (95% CI 11% to 21%), respectively, after adjusting for other prognostic clinical-pathological factors. Among patients without lymph node metastasis and TILs ≥30%, the 3-year invasive disease-free survival (iDFS) was 92% (95% CI, 89% to 98%), distant disease-free survival(D-DFS)was 97% (95% CI, 95% to 99%), and OS was 99% (95% CI, 97% to 100%). This study confirms that TILs are an independent prognostic factor in early-stage TNBC, with quantitative assessment significantly enhancing the predictive utility of conventional clinical-pathological models. Lymph node-negative patients with high TILs (≥30%) demonstrated excellent survival outcomes following standard adjuvant chemotherapy, suggesting TILs could be a biomarker for treatment stratification or clinical trial design. Additionally, a separate meta-analysis encompassed 906 patients with primary TNBC undergoing neoadjuvant chemotherapy (NACT) across six randomized trials (35). TILs were quantified as a continuous variable and categorized into three predefined groups: low (TILs: 0–10%), intermediate (TILs: 11–59%), and high (TILs: ≥60%). Within the TNBC subtype, TILs concentration was positively correlated with pCR. The pCR rate in the high TILs group was 50%, significantly higher than the 31% observed in the low TILs group. Indeed, pCR was achieved in 31% of the 80 low TILs cases, 31% of the 117 intermediate TILs cases, and 50% of the 136 high TILs cases (273 total). In univariate analysis, a 10% increase in TILs was associated with longer DFS (hazard ratio [HR]: 0.93; 95% CI: 0.87–0.98; p = 0.011) and OS (HR: 0.92; 95% CI: 0.86–0.99; p = 0.032). The disease-free survival in the high TILs group was 22.1 months, compared with 18.4 months in the low TILs group. The overall survival in the high TILs group was 40.3 months, versus 35.2 months in the low TILs group. These data demonstrate that TILs can predict response to NACT and clinical benefits.

### In untreated early-stage TNBC

Regarding the prognostic value of TILs in untreated early-stage patients with TNBC, Park et al. retrospectively demonstrated across four multicenter cohorts (encompassing 476 patients) that stromal TILs (sTILs) constitute an independent prognostic factor for OS, iDFS, and D-DFS in early-stage patients with TNBC who did not receive adjuvant chemotherapy (36). The number of sTILs was significantly associated with survival outcomes across all three endpoints. For each 10% increase in sTILs, the hazard ratio for iDFS was 0.93 (95% CI 0.87–1.00), the hazard ratio for D-DFS was 0.89 (95% CI 0.81–0.98), and the hazard ratio for OS was 0.91 (95% CI 0.82–1.00). Multivariate analysis revealed that for every 10% increase in sTILs, the hazard ratios (HRs) for iDFS, D-DFS, and OS were 0.90 (95% CI 0.83–0.98), 0.86 (95% CI 0.77–0.95), and 0.88 (95% CI 0.79–0.98), respectively, indicating sTILs as an independent prognostic factor (all P values significant). Among stage I TNBC patients with sTILs ≥30% (n=74), 5-year iDFS, D-DFS, and OS rates were 91%, 97%, and 98%, respectively, demonstrating an excellent prognosis. sTILs showed significant association with tumor grade (P<10^-^³) but were unrelated to age, tumor size, or lymph node status. These findings provide important evidence for individualized treatment decisions in TNBC and support further validation of the clinical utility of the sTIL in prospective studies. Similarly, De Jong et al. retrospectively analyzed sTILs in 441 untreated young (<40 years) N0 (lymph node-negative) patients with TNBC(90% with T1 or T2 tumors) from the Dutch Prospective Cancer Registry, distinguishing between high sTILs (≥75%) and low sTILs (<30%) groups (37). Compared with patients with low sTILs (38.4%; 95% CI: 32.1–44.6), those with high sTILs (2.1%; 95% CI: 0–5) exhibited a higher 15-year cumulative incidence of distant metastasis and death. Furthermore, each 10% increase in sTILs was associated with a 19% reduction in mortality risk (adjusted HR: 0.81; 95% CI: 0.76–0.87). The 10-year cumulative incidence of distant metastasis or death was 2.1% in high sTILs patients versus 37.0% in low sTILs patients. This retrospective analysis confirmed favorable long-term outcomes in young patients with lymph node-negative TNBC who had high sTILs (≥75%) and did not receive systemic therapy. These findings support the use of sTILs as a complementary standard clinical-pathological variable in future prospective clinical trials investigating (neo)adjuvant chemotherapy de-intensification strategies.

### In residual disease following NACT

Two studies have demonstrated a relationship between TILs in residual disease following NACT and prognosis. One study analyzed the prognostic impact of residual disease tumor-infiltrating lymphocytes (RD TILs) and residual cancer burden (RCB) following NACT in patients with TNBC. It demonstrated that RD TILs provide independent and additive prognostic information in patients with TNBC after NACT, particularly within the RCB category II (38). This study integrated data from four cohorts of TNBC patients undergoing neoadjuvant chemotherapy, including 375 individuals who did not achieve pCR. The research confirmed that elevated levels of RD TILs were associated with improved recurrence-free survival (RFS) (HR = 0.86, 95% CI 0.79–0.92, P < 0.001) and OS (HR = 0.87, 95% CI 0.80–0.94, P < 0.001), maintaining independence in multivariate analysis (RFS P = 0.032; OS P = 0.038). RD TILs provided significant incremental prognostic information for RCB staging (RFS P<0.001; OS P = 0.021), with the strongest prognostic effect observed in RCB II patients (interaction P<0.01), whereas the prognostic value of TILs was not significant in RCB III patients. TIL levels in residual TNBC lesions constitute an independent prognostic marker, potentially refining risk assessment through RCB staging, particularly for patients in RCB II. This finding aids in identifying patient subgroups likely to benefit from adjuvant chemotherapy or immunotherapy and informs the design of endpoints for future clinical trials. Another retrospective multicenter study enrolled 278 patients with triple-negative breast cancer from three European institutions. All patients had residual disease following neoadjuvant chemotherapy and had assessable tumor-infiltrating lymphocyte levels (39). Quantified lymphocyte infiltration levels are within tumors (It-TIL) and stroma (Str-TIL). The high TIL group was defined as It-TIL and/or Str-TIL > 60%, while the low TIL group was ≤60%. Univariate analysis revealed that It-TIL and Str-TIL were significantly associated with metastasis-free survival (MFS) and OS. For each 10% increase in Str-TIL, the risk of metastasis and death decreased by 21% (HR 0.79, 95% CI 0.71–0.88 for MFS; HR 0.79, 95% CI 0.71–0.89 for OS; P values < 0.001 for both outcomes). A 10% increase in It-TIL was associated with a 22% reduction in the risk of metastasis (HR 0.78, 95% CI 0.68–0.89) and a 23% reduction in the risk of death (HR 0.77, 95% CI 0.68–0.88). Multivariate analysis further confirmed the predictive value of Str-TIL and It-TIL for MFS (Str-TIL HR 0.86, 95% CI 0.77–0.96, P = 0.01; It-TIL HR 0.85, 95% CI 0.75–0.98, P = 0.02) and OS (Str-TIL and It-TIL HRs: 0.86, 95% CI 0.77–0.97, P = 0.01 and 0.86, 95% CI 0.75–0.99, P = 0.03), respectively. The 5-year MFS rate was 81.5% (95% CI 57%–93%) in the high TIL group versus 46% (95% CI 42%–55%) in the low TIL group; The 5-year OS rate was 91% (95% CI 68%–97%) in the high TIL group versus 55% (95% CI 48%–61%) in the low TIL group. In patients with lymph node-negative status and residual tumor ≤2 cm, TIL presence exhibited no prognostic association; however, in those with lymph node-positive status or residual tumor >2 cm, the high TIL group demonstrated a significant prognostic advantage. Among 27 patients with high TIL residual disease, most exhibited lower TIL levels after chemotherapy compared to pre-treatment. This suggests that chemotherapy may induce lymphocyte activation and recruitment. The study further demonstrated that post-chemotherapy TIL levels in residual disease serve as favorable prognostic indicators for patients with TNBC. This finding may represent a novel surrogate marker of therapeutic efficacy and could be utilized in the neoadjuvant setting for drug response assessment and patient stratification.

Furthermore, collated clinical trial data (**Table 1**, **Supplementary Table S1**) indicated that higher sTIL levels are consistently associated with improved PFS and OS in TNBC.

**Table 1:** Prognostic and predictive value of TILs in TNBC.

Research	Design	Population	Interventions	Results
ECOG 2197 and ECOG 1199 (40)	Two phase III, randomized trials.	481 patients with operable TNBC (from both trials).	E2197: adjuvant doxorubicin plus either cyclophosphamide or docetaxel. E1199: adjuvant doxorubicin plus cyclophosphamide followed by one of four taxane regimens.	Higher sTIL score was significantly correlated with improved DFS, distant recurrence-free survival, and OS. Results of iTIL analysis did not reach statistical significance.
Results from the FinHER trial (41)	Phase III, randomized controlled trial	1010 early-stage BC patients, 778 of whom were HER2-nonamplified.	Three cycles of docetaxel or vinorelbine, followed by (in both groups) three cycles of fluorouracil, epirubicin, and cyclophosphamide. The 232 women whose tumors had an amplified HER2/neu gene were further assigned to receive or not to receive nine weekly trastuzumab infusions.	In TNBC (n = 134) each 10% increase in TILs was significantly associated with decreased distant recurrence in TNBC; In HER2+ BC (n = 209), each 10% increase in lymphocytic infiltration was significantly associated with decreased distant recurrence.
BIG 02-98 (42)	Phase III, randomized trial.	2,887 patients with lymph node–positive BC.	Anthracycline-only (doxorubicin followed by cyclophosphamide, methotrexate and fluorouracil [CMF] or doxorubicin plus cyclophosphamide followed by CMF) versus chemotherapy combining doxorubicin and docetaxel (doxorubicin plus docetaxel followed by CMF or doxorubicin followed by docetaxel followed by CMF).	In node-positive, ER-negative/HER2-negative BC, increasing lymphocytic infiltration was associated with excellent prognosis.
IBCSG phase III randomized clinical trial 22-00 (43)	Phase III; multi-center, randomized, adjuvant phase III trial.	1081 patients with estrogen (ER) and progesterone (PgR) receptor-negative tumors, and any HER2 and nodal status(following breast cancer surgery and standard induction chemotherapy).	Low-dose CM-maintenance (cyclophosphamide and methotrexate) and no maintenance chemotherapy (No-CM).	TILs score is a potent prognostic factor in patients with TNBC. Low-dose chemotherapy confers a greater (not statistically significant) clinical benefit in patients with LPBC(lymphocyte predominant breast cancer).
Prognostic and predictive value of tumor-infiltrating lymphocytes in two phase III randomized adjuvant breast cancer trials (44)	Two phase III; randomized controlled trial.	1146 patients were included: 311 high-risk node-negative premenopausal patients and 835 high-risk node-negative or node-positive postmenopausal patients.	Six courses of 5-fluorouracil, doxorubicin or epirubicin and cyclophosphamide or no chemotherapy.	Confirmed the prognostic role of TIL in triple-negative early breast cancer and suggested a prognostic impact in HER2+ patients as well. TIL should not be used as a parameter to select patients for anthracyclines chemotherapy.
KEYNOTE-173 (45)	Phase Ib trial	60 patients with early-stage TNBC.	Neoadjuvant pembrolizumab + chemotherapy.	Median pretreatment sTIL levels were higher in patients who achieved pCR than in those who did not (42% vs. 10%).
CALGB 40502 (46)	Phase III, randomized trial.	799 patients with advanced (stage IIIC or IV) breast cancer.	First-line nab-paclitaxel, ixabepilone, or paclitaxel, with or without bevacizumab.	Low sTIL scores were significantly associated with worse PFS (HR 1.34) and OS (HR 1.32). When controlled for hormone receptor status, the trend was similar but did not reach statistical significance.
BELLINI (47)	Phase II, adaptive trial.	46 patients with early-stage TNBC.	Neoadjuvant nivolumab (with or without ipilimumab).	High pretreatment TILs were associated with improved response to immunotherapy. Responders had shorter CD8+ to tumor distance.
KEYNOTE-086 (48)	Phase II, singlearm trial.	254 patients with metastatic TNBC.	Pembrolizumab monotherapy after progression on one or more systemic therapies.	Patients with high sTILs (>/= median amount within the sample of patients) had increased ORR (odds ratio 1.26)
KEYNOTE-119 (49)	Phase III, randomized trial.	622 patients with previously treated metastatic TNBC.	Pembrolizumab monotherapy vs. single-agent chemotherapy.	High sTIL scores were significantly associated with improved OS, ORR, PFS, and duration of response in the pembrolizumab arm, but not the chemotherapy arm.
A randomized phase II study investigating durvalumab in addition to an anthracycline taxane-based neoadjuvant therapy in early triple-negative breast cancer: clinical results and biomarker analysis of GeparNuevo study (50)	Phase II, randomized trial.	117 patients with metastatic TNBC.	Nab-paclitaxel followed by epirubicin and cyclophosphamide, plus durvalumab or placebo given every 4 weeks.	sTILs as a continuous variable were significantly associated with improved response rates in both treatment arms.
A non-inferiority, phase III trial of gemcitabine plus capecitabine versus gemcitabine plus carboplatin as first-line therapy and tumor-infiltrating lymphocytes as a prognostic biomarker in patients with advanced triple-negative breast cancer (51)	Phase III; randomized trial	187 patients who had not previously received systemic therapy for advanced TNBC.	Gemcitabine plus oral capecitabine or gemcitabine plus carboplatin.	The GC regimen showed better efficacy compared with the GX regimen in patients with high CD8+ TILs. However, the GX regimen should be considered in patients who cannot tolerate hematological toxicity.

## Clinical translation strategies for TIL therapy

**Figure 2:**
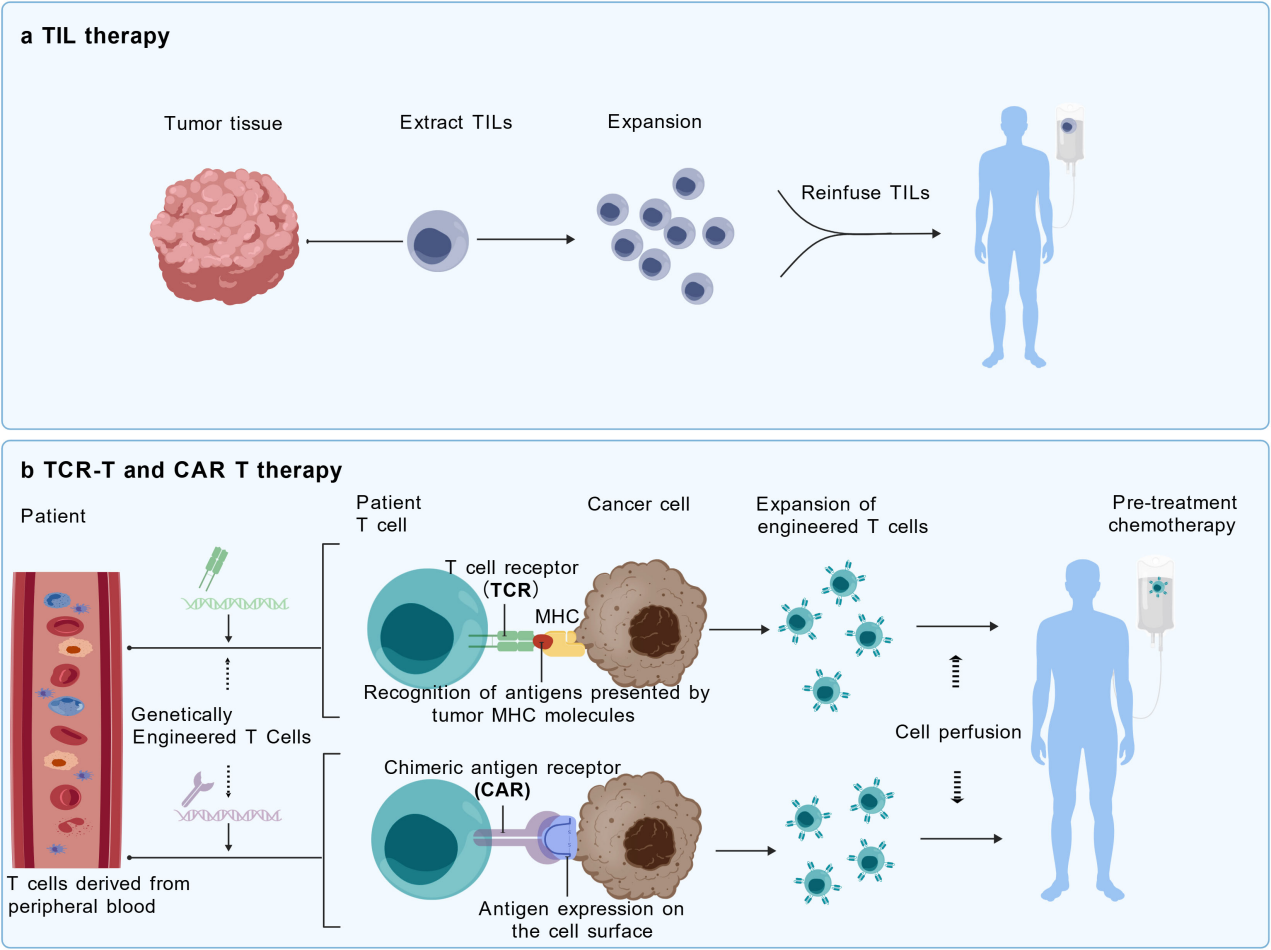
**(a)** TIL therapy involves isolating TILs from the patient's tumor tissue—lymphocytes that have naturally infiltrated the tumor microenvironment and exerted cytotoxic effects. After *in vitro* expansion, these TIL cells are reinfused into the patient to continue their antitumor activity. **(b)** In contrast, TCR-T and CAR-T cell therapies involve genetically modifying the patient's own T cells *in vitro* before reinfusion. CAR-T relies on artificially engineered single-chain antibody fragments (Chimeric antigen receptor; CARs) that recognize tumor surface antigens. These CARs transmit signals through intracellular co-stimulatory molecules to activate T cells, representing synthetic molecules. TCR-T cells, on the other hand, are engineered through gene editing to introduce T cell receptor (TCR) genes that can specifically recognize tumor antigens presented by tumor MHC molecules. This enables them to express exogenous TCRs, thereby gaining the ability to selectively kill tumor cells. TCR-T cells more closely resemble the body's natural T cells. They primarily rely on affinity-optimized or naturally occurring TCRs to recognize antigens presented by tumor MHC molecules, transmitting stimulatory signals intracellularly via the TCR-CD3 complex. CAR-T therapy has achieved significant success in treating hematologic malignancies, but breakthroughs in solid tumor therapy remain elusive. TIL therapy offers unique advantages for treating solid tumors, although its efficacy and safety require further validation. TCR-T therapy demonstrates immense potential for broader target recognition and solid tumor treatment.

Adoptive cell transfer therapy with TIL (ACT-TIL) is a personalized immunotherapy that involves isolating lymphocytes from the patient’s tumor tissue, expanding and activating them *in vitro*, and then reinfusing them into the patient to attack tumor cells (52). ACT with TILs was first demonstrated and applied by Rosenberg et al. in melanoma treatment, yielding satisfactory results (53). Unlike CAR-T therapy, this form of ACT primarily sources TILs from the tumor tissue of the patients. These TILs are extracted and selected for their ability to secrete cytokines, cultured *in vitro*, and then reinfused into the patient (54). Not being a specific antigen-targeted therapy and using naturally selected tumor-reactive lymphocytes, TIL therapy may theoretically overcome the problems of antigenic heterogeneity, tumor trafficking, and on-target off-tumor toxicity that limit the effectiveness of CAR-T and TCR-T therapies against advanced solid tumors. **Figure 2** compares the mechanisms and clinical differences of various ACT therapies.

T cells that recognize multiple cancer cell surface antigens are key to the combined ACT and TILs attacking tumors. In a prior study, 10 out of 13 patients with metastatic melanoma who had failed other treatment regimens received an infusion of the TIL product. Among these 10 patients, 50% achieved clinical efficacy, comprising two complete and three partial responses. Notably, neoantigen-specific T cell populations emerged following TIL infusion and persisted in the peripheral blood of all 10 patients (55). The first autologous TIL cell therapy, lifileucel, was recently approved by the U.S. Food and Drug Administration for the treatment of adult patients with unresectable or metastatic melanoma who have previously received PD-1 inhibitor antibody therapy. Beyond melanoma, it is not well established whether metastatic lesions treated with an immune checkpoint inhibitors (ICIs) with or without cytotoxic chemotherapy can reliably serve as manufacturing sources for TIL cell therapy for clinical use or whether patients with other tumor types could tolerate and respond to the TIL cell therapy regimen (56). TILs have yielded consistent outcomes in treating other solid tumors. However, some Phase I/II clinical trials have demonstrated promising efficacy data in settings such as non-small cell lung cancer(NSCLC), cervical carcinoma, and head and neck squamous cell carcinoma (57). Studies indicate that TILs expanded *in vitro* from various solid tumors comprise oligoclonal effector T cells that exhibit reactivity towards a heterogeneous repertoire of tumor-associated antigens (58). A clinical trial demonstrated that TIL-based ACT combined with rapidly expanded *in vitro* TILs (REP TILs) and ICIs is feasible across multiple solid tumors beyond melanoma. Clinical efficacy and tumor regression were confirmed in a subset of patients, supported by the *in vitro* antitumor reactivity of REP TILs, whose phenotype correlated with clinical response (59). In this study, TILs were established using irradiated allogenic feeder cells, anti-CD3 antibody, and IL-2 in a rapid expansion protocol (REP). The production of REP TILs has been described in detail previously (60). A clinical trial on NSCLC indicates that many patients exhibit poor or no response to ICI monotherapy (primary or secondary resistance). Combination therapy with TILs, by introducing large numbers of fully functional T cells, holds the potential to circumvent or overcome such resistance and reignite effective antitumor immune responses (61). Furthermore, breast cancers can express naturally processed and presented unique nonsynonymous mutations that are recognized by a patient’s immune system. TILs recognizing these immunogenic mutations can be isolated from a patient’s tumor, suggesting that adoptive cell transfer of mutation-reactive TILs could be a viable treatment option for patients with breast cancer (62). Owing to the standard treatment regimens (such as chemotherapy and radiotherapy) being ineffective in TNBC, whereas combining ACT with TILs effectively addresses this issue. Zacharakis selected forty-two patients with metastatic breast cancer who received concurrent injections of short-term cultured TILs and pembrolizumab. Three out of six patients demonstrated efficacy, with one patient with TNBC achieving complete regression of liver, lymph node, and mediastinal metastases after sixty-six months of treatment. Subsequent disease control was achieved using surgery alone (63). Furthermore, ACT combined with TIL therapy for TNBC is currently recruiting volunteers (NCT04842812 and NCT04111510), fully demonstrating the immense potential of this treatment. Pooled clinical trial data are adapted from clinicaltrials.gov and listed in **Table 2**.

**Table 2:** Clinical trials based on TIL-mediated therapy.

Identifier	Phase	Status	Intervention/Treatment
NCT04111510	II	Completed	LN-145 will be delivered as a single therapy in patients with Metastatic Triple Negative Breast Cancer. Intravenous interleukin-2 (IL-2) administrations for up to six doses maximum (during inpatient hospitalization).
NCT01174121	II	Recruiting	All patients will receive a non-myeloablative, lymphodepleting preparative regimen consisting of cyclophosphamide and fludarabine followed by the infusion of autologous TIL and high-dose aldesleukin.
NCT04842812	II	Recruiting	Grow TILs and engineered the tumor-effective TILs with CRISPRA-CAS9 technique to knockdown PD1 and electronic-transfection strategy to express scFvs that target PD1 and CTLA4; amplify the engineered T cells as needed, test the quality and killing activity of the TILs and then transfuse them back the patients via systemic or local injections via standard protocol.
NCT05451784	I/II	Recruiting	Performing a tumor biopsy and selecting isolation and expansion of PD1-postive TILs to manufacture the final product NUMARZU-001. PD1^+^ TILs (NUMARZU-001) product infusion with auxiliary medication (NMA-LD chemotherapy or IL-2).
NCT05142475	II	Recruiting	Autologous TILs are expanded from tumor resections or biopsies. 1x10^9-5x10^10 *in vitro* expanded autologous TILs will be infused iv. to patients with advanced breast cancer after NMA lymphodepletion treatment with hydroxychloroquine and cyclophosphamide.
NCT06107894	I	Not yet recruiting	Begin with intravenous non-myeloablative (NMA) lymphodepleting regimen composed by cyclophosphamide and fludarabine, followed by infusion of autologous TILs (NEOG-100). Patients in Cohort 1 will receive NEOG-100 and patients in Cohort 2 will receive NEOG-100 plus low-dose (2 MIU) IL-2.
NCT06532812	I/II	Recruiting	TILs will be harvested from patients’ tumors, expanded *in vitro*, and infused back into the patients following a non-myeloablative lymphodepletion regimen. Pembrolizumab, a monoclonal antibody that targets the PD-1 receptor on T cells, will be administered to enhance the immune response.
NCT05576077	I b	Terminated	TBio-4101 is an autologous tumor infiltrating lymphocyte (TIL) therapy that utilizes tumor specific antigens to select, sort, and expand patient-specific tumor-reactive T-cells to be reinfused into the patient. The adoptive cell therapy is further enhanced through the use of non-myeloablative chemotherapy prior to TIL infusion, followed by the TIL plus IL-2 infusion. Low-dose radiation therapy is administered prior to and after TIL plus IL-2 infusion. Pembrolizumab is provided after the resolution of IL-2 toxicities.

## Clinical challenges and countermeasures for TIL Therapy in TNBC

Despite the encouraging results of TIL therapy in clinical trials, numerous practical and economic challenges have limited its widespread implementation (64). To date, TIL therapy has been evaluated in younger patients with an ECOG performance status(Eastern Cooperative Oncology Group Performance Status, ECOG PS, PS) of 0 to 1. Even within this selected cohort from clinical trials, it is frequently observed that only a small proportion of screened patients ultimately qualify for treatment. Standardized TIL assessment optimizes individualized prognosis prediction and provide a basis for formulating immunotherapy strategies. Future studies should explore the potential of TIL-guided treatment de-escalation or immunotherapy combination regimens in high-risk patients (65).

Current TIL amplification methods are influenced by multiple factors, including tumor size, TIL abundance within tumor tissue, and susceptibility to contamination, resulting in over 60% of patients being unable to yield autologous TILs. The prolonged *in vitro* amplification time and low success rates further limit the clinical application of TILs. Traditional TIL expansion relies on surgically resected tumor specimens. Sample acquisition is constrained in patients with inoperable or small lesions. Research indicates that tissues obtained through CNB from primary TNBC sites can be used for TILs expansion. The expanded TILs exhibit comparable expansion capacity, phenotype, and cytokine secretion profiles to those derived from surgical specimens, offering new possibilities for inoperable patients (66).

The *in vitro* expansion of TILs in TNBC typically comprises three stages: TIL collection from tumor tissue, preliminary amplification during the pre-expansion phase, and rapid amplification. Existing methods require approximately 8–10 weeks to complete the three-stage TIL expansion process. This excessively prolonged *in vitro* culture period also leads to TIL exhaustion, significantly diminishing the therapeutic efficacy against the tumors (67). Standard expansion protocols involve adding IL-2 to the culture medium. Prolonged co-culture of TILs with high concentrations of IL-2 reduces the percentage of CD8+ T cells percentage, ultimately impairing the anti-tumor activity of TILs (68). Conversely, generating T cells with central or stem cell memory properties *in vitro* holds greater potential for antitumor immunity (69, 70), high concentrations of IL-2, whilst capable of stimulating T-cell expansion, may also induce terminal differentiation of T cells, shortening their proliferative lifespan and reducing the proportion of stem-like memory T cells (Tscm), thereby compromising persistence following reinfusion. Previous studies have indicated that priming T cells with common gamma chain cytokines, IL-7, IL-15, or IL-21, can generate and sustain stem-like memory T cells, demonstrating promise in preclinical models of adoptive T cell therapy (71). Consequently, it enables more effective simulation of the *in vivo* environment and obtain higher-quality TIL products by introducing other γ-cytokine molecules.

TCR, co-stimulatory molecules, and cytokines mediate numerous internal cascades, facilitating T cell differentiation. These signals promote immunological memory; however, they may also drive T cell differentiation and exhaustion (72). Research indicates that inhibiting signaling pathways may enhance T cell therapy. Increasing glucose transporter expression and glycolytic activity is one of the key mechanisms by which co-stimulatory receptor stimulation promotes T cell growth (73). The PI3K/ATK/mTOR signaling axis is crucial for reconnecting metabolism, promoting growth, protein translation, and function in all proliferating cells. Consequently, employing compounds that target components of this pathway represents a judicious approach to modulating T cell biology and enhancing its antitumor potential (74). Experiments have demonstrated that targeting the lymphocytes-specific delta subunit of PI3K generates a less differentiated state. Co-administration of a PI3Kδ inhibitor and a vasoactive intestinal peptide antagonist enhances the *in vitro* expansion, *in vivo* persistence, and anti-cancer cytotoxicity of these T cells (75, 76). Research indicates that inhibition of the rapamycin target complex 1 (mTORC1) in activated human immature T cells by either rapamycin or the Wnt-β-catenin signaling activator TWS119 induces TSCM cells. Pharmacologically induced TSCM cells exhibit superior functional characteristics following adoptive transfer, demonstrating sustained proliferative capacity (77). Further studies have indicated that antigen-activated CD8+ T cells treated with rapamycin generate five times more long-lived memory T cells *in vivo* than untreated control T cells. Raptamycin-treated T cells demonstrated superior survival capacity compared to control T cells. These cells may represent a unique cellular model for identifying nutrients and signals critical for regulating the metabolic processes of effector T cells and memory T cells, and for developing novel approaches to enhance the efficacy of adoptive T cell cancer therapies (78). These findings reveal an intriguing approach to modulate T cell differentiation and metabolism, enhancing their tumor-killing capabilities. Furthermore, Gurusamy et al. used multi-phenotypic screening to identify the phenotype of effective antitumor T cells, revealing p38 kinase as a central regulator of therapeutically required T cell characteristics. p38 kinase inhibition promotes effective T cell phenotypes and enhances the antitumor efficacy of adoptive T cell immunotherapy (79).

Some studies have indicated that modifying the *in vitro* nutritional environment to induce beneficial metabolic adaptations can enhance the capacity of expanded T cells to delay tumor growth. Increasing glycolytic flux drives CD8+ T cells toward a terminally differentiated state, while inhibiting glycolysis maintains the formation of long-lived memory CD8+ T cells. This has significant implications for improving the efficacy of T cell-based therapies against chronic infectious diseases and cancer (80). During transient glucose deprivation, a window of opportunity exists for the revitalization of effector CD8+ T cells. Research indicates that brief glucose depletion following activation induces metabolic reprogramming in effector CD8+ T cells, thereby enhancing their *in vivo* longevity and functional capacity (81). Research findings indicate that T cells with elevated L-arginine levels exhibit superior antitumor activity. Supplementation with L-arginine *in vitro* and *in vivo* enhances T-cell tumor-killing capacity by promoting memory formation and mitochondrial respiration. The beneficial effects of L-arginine on T cell survival and antitumor function may be harnessed therapeutically, such as enhancing adoptive T cell therapy (82).

Overall, TIL therapy offers certain unique advantages in treating solid tumors; however, it continues to face several challenges and limitations. The tumor-suppressive microenvironment remains the primary obstacle to TIL treatment. Considerable room for improvement still exists in isolating and expanding effective tumor-reactive T cells, while alternative combination therapies require further exploration.

## Conclusion

TILs play a pivotal role in the tumor immune microenvironment of TNBC, with their quantity, composition, and spatial distribution carrying significant biological and clinical value. Traditional visual assessment is being revolutionized and supplemented by digital, automated AI methods, consequently driving the widespread application of TILs as reliable biomarkers for TNBC prognosis stratification, treatment decision-making, and efficacy prediction. This ultimately facilitates the realization of more precise and individualized therapies. These technological advances collectively propel TILs as a reliable biomarker for widespread application in TNBC prognosis stratification, treatment decision-making, and efficacy prediction, ultimately facilitating more precise personalized therapy.

TIL therapy offers a promising new therapeutic strategy with significant potential for individualized treatment for patients with breast cancer, particularly those with TNBC and specific HER2-positive breast cancer lacking effective treatment options. It represents a significant exploration in tumor immunotherapy, shifting from a “broadly immunological” approach towards “deep personalization”. Although currently primarily in clinical trial phases and facing multiple challenges, including technical, accessibility, and efficacy optimization issues, TIL therapy is expected to gradually achieve clinical adoption in the coming years with ongoing technological advancements and accumulating clinical data. This may even transform the treatment landscape for certain advanced breast cancers.
